# The Sac3 TPR-like region in the *Saccharomyces cerevisiae* TREX-2 complex is more extensive but independent of the CID region

**DOI:** 10.1016/j.jsb.2016.07.007

**Published:** 2016-09

**Authors:** Shintaro Aibara, Xiao-Chen Bai, Murray Stewart

**Affiliations:** MRC Laboratory of Molecular Biology, Francis Crick Avenue, Cambridge Biomedical Campus, Cambridge CB2 0QH, United Kingdom

**Keywords:** TREX-2, transcription-export complex-2, mRNA nuclear export, TREX-2 complex, Cryo-EM, Crystallography

## Abstract

Transcription-export complex 2 (TREX-2 complex) facilitates the localization of actively transcribing genes to the nuclear periphery and also functions to contribute to the generation of export-competent mRNPs through interactions with the general mRNA nuclear export factor Mex67:Mtr2. The TREX-2 complex is based on a Sac3 scaffold to which Thp1, Sem1, Cdc31, and Sus1 bind. TREX-2 can be subdivided into two modules: one, in which Thp1 and Sem1 bind to the Sac3^M^ region (residues ∼100–551), and the other in which Cdc31 and two Sus1 chains bind to the Sac3^CID^ region (residues ∼710–805). Complementary structural analyses using X-ray crystallography, electron microscopy, and small-angle X-ray scattering of the *Saccharomyces cerevisiae* TREX-2 complex, expressed using Baculovirus-infected Sf9 cells, have indicated that the TPR-like repeats of the Sac3^M^ region extend considerably further towards the N-terminus than previously thought, and also indicate that this region and Sac3^CID^:Sus1:Cdc31 region of the *S. cerevisiae* complex are structurally independent. Although the density visible accounted for only ∼100 kDa, a 5.3 Å resolution cryo-EM reconstruction was obtained of the M-region of TREX-2 that showed an additional three putative α-helices extending towards the Sac3 N-terminus and these helices were also seen in a 4.9 Å resolution structure obtained by X-ray crystallography.

**Summary statement:**

We describe the expression, purification and structural characterization of the *S. cerevisiae* TREX-2 complex and demonstrate that the Sac3 TPR-like repeats are more extensive than previously thought and that the M- and CID-regions do not appear to have a defined spatial orientation.

## 1 Introduction

**Fig. 1:**
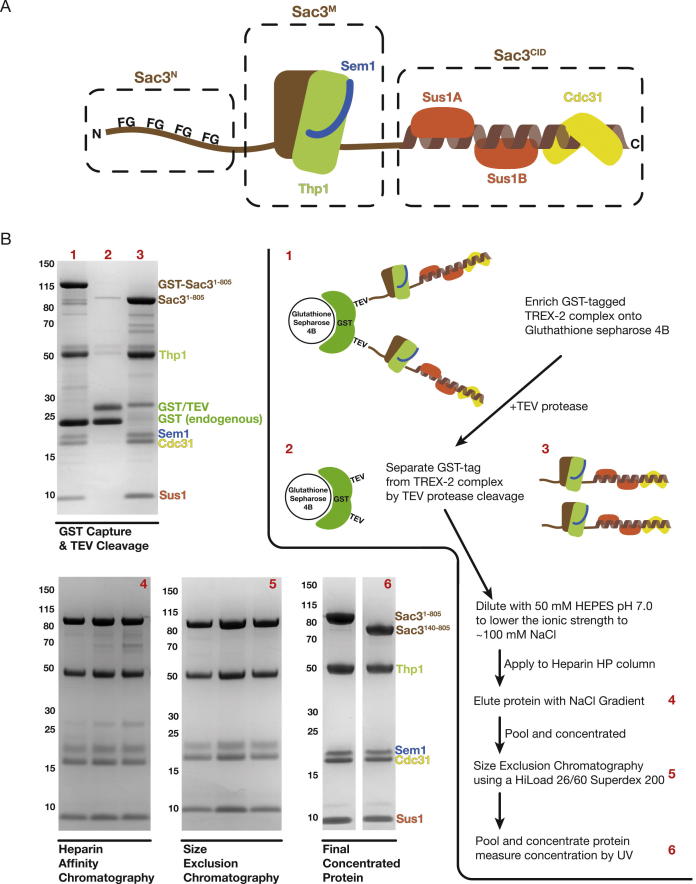
(A) Schematic representation of the TREX-2 complex that is formed around a Sac3 scaffold (brown). The Sac3 scaffold protein can be broadly split into three sections whereby the most N-terminal region of Sac3 (labeled Sac3^N^) and its homologues (such as GANP from *H. sapiens*) contain FG-nucleoporin-like motifs. The middle region of Sac3 (labeled Sac3^M^) forms a complex with Thp1 and Sem1 to form a nucleic acid binding platform formed by its winged-helix domains, and the C-terminal region of Sac3 (labeled Sac3^CID^) interacts with one Cdc31 chain (yellow) and two Sus1 chains (red) in *S. cerevisiae*. (B) Flowchart for the purification scheme established for producing recombinant TREX-2 complex and the corresponding gels. Numbers in red match the purification step and the Coomassie-stained SDS gel.

The TREX-2 complex (Transcription-Export complex 2) is conserved across eukaryotes and integrates mRNA export into the gene expression pathway and, in yeast, also mediates the location of actively-expressed genes such as *GAL1* to the nuclear envelope (García-Oliver et al., 2012, Köhler and Hurt, 2007, Rodríguez-Navarro et al., 2004). The TREX-2 complex is based on a Sac3 scaffold to which Thp1, Sem1, Cdc31, and two copies of Sus1 bind (Fig. 1A) and broadly speaking, the TREX-2 complex can be subdivided into three regions: the Sac3 N-terminus (Sac3^N^; residues 1–100), which harbors degenerate FG-like repeats similar to those seen in many nuclear pore proteins (FG nucleoporins) (Fischer et al., 2002); the M-subcomplex, consisting of Sac3 residues ∼100–551 bound to Thp1 and Sem1, which forms a nucleic acid binding module as well as docking site for components of the Mediator complex (Ellisdon et al., 2012, Schneider et al., 2015, Valkov and Stewart, 2015); and the CID-subcomplex, consisting of Sac3 residues ∼720–805 bound to Cdc31 and two Sus1 chains and which, in *Saccharomyces cerevisiae*, binds to the nuclear pore complex (NPC) to tether the complex close to the nuclear basket to facilitate localization of genes such as *GAL1* (Jani et al., 2014, Jani et al., 2009).

The TREX-2 complex has been implicated in a spectrum of biological roles including preventing genome instability, transcription initiation, gene-gating, and mRNA export (Bhatia et al., 2014, Faza et al., 2009, Gallardo et al., 2003). Previous work using homologues from the thermophile *Chaetomium thermophilum* indicated that the multiple FG-like repeats present on the Sac3 N-terminal region may serve as a docking site for the principal mRNA export factor Mex67:Mtr2 as well as making intra-molecular interactions with the CID-subcomplex to form an annular structure (Dimitrova et al., 2015). Although the FG-repeats in the *S. cerevisiae* Sac3 N-terminal region are fewer and less similar to nucleoporin FG repeats, deletion of residues 1–140 generates growth and mRNA export defects (Dimitrova et al., 2015).

Biochemical and structural studies of the entire *S. cerevisiae* TREX-2 complex has been frustrated because of the low native abundance of Sac3 as well as problems encountered with its expression in bacteria or yeast systems. For example, the overexpression of *S. cerevisiae* Sac3 in yeast cells is lethal, due to the sequestration of Cdc31 causing defects in cell division (Fischer et al., 2002, Jani et al., 2009). Consequently, investigations have focused on subcomplexes of the TREX-2 complex, based on the Sac3 N-terminal region, the M-subcomplex, and a range of CID-complexes (Dimitrova et al., 2015, Ellisdon et al., 2012, Jani et al., 2009). A hybrid expression approach was employed to purify the TREX-2 complex from *C. thermophilum* where Sac3, Thp1, and Sem1 were co-expressed in *S. cerevisiae* to which pre-purified Cdc31 and Sus1 from *Escherichia coli* were added, but this was only possible because the *C. thermophilum* TREX-2 components were sufficiently different from the *S. cerevisiae* machinery (Dimitrova et al., 2015) to impair sequestration of Cdc31.

To obtain sufficient quantities of the *S. cerevisiae* TREX-2 complex for structural studies, we have overexpressed its five components simultaneously in Baculovirus-infected sf9 insect cells. This material has then been investigated using spectrum of complementary structural techniques including X-ray crystallography, electron microscopy, and small-angle X-ray scattering to demonstrate that the TPR-like repeats extend further towards the Sac3 N-terminus than previously thought. It is anticipated that this capability to express and purify the entire TREX-2 complex from *S. cerevisiae* will allow further biochemical and biophysical investigations to be conducted, whereas the more extensive TPR-like repeats of Sac3^M^ may contribute to interaction with both RNAs and the mediator complex.

## 2 Methods

### 2.1 Cloning

Bacmids encoding for the entire TREX-2 complex were produced using a modified *Multi*Bac approach (Bieniossek et al., 2008). Sac3, Thp1, Sem1, Cdc31, and Sus1 were initially cloned individually into separate vectors to flank them with the polH promoter and SV40 terminator. Cassettes including the promoter, gene, and terminator were then combined using Gibson Assembly (Gibson et al., 2009). This was repeated iteratively to produce one plasmid with the five gene cassettes in tandem.

### 2.2 Baculovirus-insect cell expression

Plasmids containing the TREX-2 components were transformed into *E. coli* DH10 EmBacY using standard heat shock procedures. Colonies in which recombination had generated bacmids harboring the gene of interest were selected by blue/white screening. These bacmids were extracted and purified using standard procedures and transfected into 2 ml of Sf9 cells (5 × 10^5^ cells per millilitre) using the FuGENE HD Transfection Reagent (Promega). P1 virus was harvested after 72 h and used to infect 100 ml of Sf9 cells (1 × 10^6^ cells per millilitre) that were then incubated at 27 °C with shaking at 127 rpm for 72 h. P2 virus was harvested by centrifugation and used to further infect 500 ml of Sf9 cells (1 × 10^6^ cells per millilitre) using 10 ml of virus per litre of cells. Sf9 cells infected with P2 were then harvested by centrifugation at 3500*g* for 30 min, flash-frozen in liquid nitrogen and stored at −80 °C until required.

### 2.3 Protein purification

Frozen pellets of Sf9 cells expressing the TREX-2 complex were thawed and resuspended in 50 mM Tris pH 8.0, 500 mM NaCl, 5 mM DTT after which the cells were lysed by gentle homogenization using 25 strokes of a Dounce manual homogenizer. The lysate was clarified twice by centrifugation at 20,000 rpm in a JA-25.50 rotor for 30 min to completely eliminate cell debris, after which the supernatant was incubated with glutathione Sepharose 4B beads (GE Healthcare) for 1 h. The beads were pelleted by gentle centrifugation at 1500*g* for 5 min and washed with 150 ml of 50 mM Tris pH 8.0, 500 mM NaCl, 5 mM DTT in a gravity-flow column. The washed and pelleted beads were then resuspended in 6 ml of 50 mM Tris pH 8.0, 500 mM NaCl, 5 mM DTT to which 1 mg of TEV protease (purified in-house (Blommel and Fox, 2007)) was added and incubated for 16 h at 4 °C with agitation to liberate the TREX-2 complex from the GST-tag.

The flow-through containing the TREX-2 complex was collected and the resin washed with 2 ml of 50 mM Tris pH 8.0, 500 mM NaCl, 5 mM DTT. To reduce the ionic strength of the buffer for the next step, 40 ml of 50 mM HEPES pH 8.0 was added to the protein before rapidly applying the sample to a Heparin HP column (GE Healthcare) pre-equilibrated in 50 mM HEPES pH 8.0, 100 mM NaCl. The column was washed with 15 column volumes of 50 mM HEPES pH 8.0, 100 mM NaCl, before running a linear gradient to 50 mM HEPES pH 8.0, 1 M NaCl. Fractions containing TREX-2 complex were analyzed by SDS-PAGE, pooled and concentrated using an Amicon concentrator to a final volume of approximately 8 ml.

The pooled and concentrated sample was then subjected to size exclusion chromatography using a Superdex S200 26/60 column pre-equilibrated in 20 mM HEPES pH 8.0, 300 mM NaCl, 5 mM DTT. Fractions containing homogeneous TREX-2 complex as assessed by SDS-PAGE were pooled and concentrated using an Amicon concentrator to a final concentration of 7 mg/ml as assessed by UV spectroscopy.

### 2.4 Negative-stain electron microscopy

Aliquots of 3 μl of purified TREX-2 complex at a concentration of ∼100 nM were placed on freshly glow-discharged carbon-coated grids and incubated for 1 min. Excess liquid was blotted away and ∼20 μl of 1% (w/v) uranyl acetate solution was applied immediately. A small amount of the stain solution was allowed to incubate on the grid for 1 min, then blotted away and dried. 1-s exposure images were recorded manually on a TecnaiT12 (FEI) electron microscope operating at 120 kV and equipped with a CCD detector.

### 2.5 Electron cryo-microscopy

Aliquots of 3 μl of purified TREX-2 complex at a concentration of ∼75 nM were placed on holey carbon grids (Quantifoil Au R1.2/1.3, 300 mesh) pre-coated with graphene oxide (Bokori-Brown et al., 2016, Pantelic et al., 2010), and flash frozen in liquid ethane using a manual plunger. Zero-energy-loss images were recorded manually on an FEI Titan Krios electron microscope at 300 kV, using a slit width of 20 eV on a GIF-Quantum energy filter. A Gatan K2-Quantum detector was used in super-resolution counting mode at a calibrated magnification of ×35,714 (yielding a pixel size of 1.43 Å). The total dose on the specimen was 40 e^−^/Å^2^ fractionated into 20 movie frames over 16 s (a dose rate of 2.5 e^−^/Å^2^/s). Defocus values in the final data set ranged from 0.8 to 4.5 μm.

### 2.6 Image processing

Cryo-EM images, were processed using MOTIONCORR (Li et al., 2013) for whole-frame motion correction, GCTF (Zhang, 2016) was used for estimation of the contrast transfer function parameters, and RELION-1.4 (Scheres, 2012) for all other image processing steps. Templates for reference-based particle picking were obtained from 2D class averages that were calculated from a manually picked subset of the micrographs using e2boxer (Tang et al., 2007). Using low-pass filtered templates to 20 Å resolution to limit reference bias, ∼162,000 particles were picked automatically from a total of 496 micrographs. Because the picking procedure is prone to false positives (Scheres, 2015), a reference-free 2D class averaging was used to select 81,559 particles for 3D refinement. After per-particle motion correction and radiation-damage weighting (particle polishing (Scheres, 2014)), these particles gave a reconstruction with a resolution of 5.3 Å. Reported resolutions are based on the gold-standard FSC = 0.143 criterion, and FSC curves were corrected for the effects of a soft mask on the FSC curve using high-resolution noise substitution (Chen et al., 2013). All 3D refinements were started from a 60 Å low-pass filtered initial model, the first of which was made from the crystal structure of the Sac3:Thp1:Sem1 (PDB accession code 3T5V). Before visualization, all density maps were corrected for the modulation transfer function of the detector, and then sharpened by applying a negative B-factor that was estimated using automated procedures (Rosenthal and Henderson, 2003). Because of the limited resolution, the new helices identified were only modelled as ideal poly-Ala helices and only rigid body refinement was employed to generate the final model. Negatively-stained images were processed in the same manner, except for the skipping of whole-frame motion correction and applying phase-flips in the stage of CTF estimation.

### 2.7 X-ray crystallography

Crystals of *sc*TREX-2^ΔC^ were obtained by sitting drop vapor diffusion using 200 nl of 20% PEG 3350, 0.2 M Mg formate that was mixed with 200 nl of *sc*TREX-2^ΔC^ at 16.5 mg/ml. Crystals grew over a period of 2 weeks and were harvested, cryo-protected using the mother liquor supplemented with 20% glycerol, and then vitrified by plunging into liquid nitrogen. X-ray diffraction data were collected on beamline I24 at the Diamond Light Source (Didcot, UK). Reflections were indexed and integrated using *XDS* (Kabsch, 2010) and then scaled and merged in *AIMLESS* (Evans, 2006, Evans and Murshudov, 2013). An initial structural model was obtained by molecular replacement using *Phaser* (McCoy et al., 2007) using the structure of *sc*Sac3^M^:Thp1:Sem1 (Ellisdon et al., 2012; PDB accession code 3T5V). Iterative cycles of rebuilding using *Coot* (Emsley et al., 2010) and refinement using *PHENIX* (Adams et al., 2010) were used to generate the final model. Because the resolution was limited to 4.9 Å, refinement was constrained using PDB:3T5V as a reference with the B-factors initially set to the Wilson B-factor and then refined as group B-factors using TLS treating each chain as a single unit.

### 2.8 Small-angle X-ray scattering

Small angle X-ray scattering (SAXS) was performed using an on-line HPLC system (Viscotek) equipped with Superdex 200 Increase 3.2/300 column (GE Healthcare) mounted on beamline BM29 at the European Synchrotron Radiation Facility (ESRF, Grenoble, France). Data collection was conducted at 20 °C, using a wavelength of 0.995 Å. Data were processed automatically by the on-line pipeline AUTOSUB (part of the ATSAS package (Konarev et al., 2003)). Pair distance distribution functions of the particles *P*(*r*) and the maximum sizes *D*_max_ were computed using GNOM (Svergun, 1992) and molecular weights were estimated by comparison of the extrapolated forward scattering, *I*(0), of the samples obtained using Guinier analysis by AUTORG (Konarev et al., 2003) with that of a bovine serum albumin standard (Sigma–Aldrich).

## 3 Results

### 3.1 Expression and purification of the entire scTREX-2 complex

Because Sac3 is expressed poorly in bacteria and is present in only small quantities in yeast where its overexpression is lethal (Fischer et al., 2002), it has been difficult to obtain sufficient quantities of the *S. cerevisiae* TREX-2 complex for structural studies. We circumvented these difficulties by expressing the *S. cerevisiae* TREX-2 complex using a single Baculovirus harboring the cDNA coding sequences of one copy of Sac3, Thp1, Sem1, Cdc31, and Sus1. The Sac3 protein was expressed as a TEV-cleavable N-terminal GST fusion, and the entire complex was co-enriched using glutathione Sepharose 4B beads. Although there appeared to be a substantial amount of endogenous GST protein from sf9 cells that also bound onto the resin, the protein could be eluted specifically by TEV protease cleavage and purified by sequential chromatography steps (Fig. 1B, gels 1–6). Coomassie-stained SDS-PAGE analysis indicated that a single peak with all five proteins present was obtained after gel filtration chromatography (Fig. 1B, gel 6). Although it was possible to generate material using full-length Sac3 (residues 1–1301), the Sac3 in these complexes was labile and was rapidly proteolyzed in its C-terminus (Supplementary Fig. 1A). Therefore efforts were focused on a construct of Sac3 extending as far as the end of the Cdc31 binding region (residue 805, TREX-2^1–805^) that displayed superior stability compared to the full length Sac3.

The identities of the proteins were confirmed by mass spectrometry and no contaminating homologous proteins from sf9 cells were identified. Minor phosphorylation of Sac3 was observed, which is commonly seem with proteins overexpressed in sf9 cells. Mass spectrometry identified residues Ser71, Ser102, Ser592, Ser600, Ser684 within Sac3 that had peptides with heavier masses consistent with phosphorylation. No other modifications of Sac3 or the other components of the TREX-2 complex were observed.

### 3.2 Structure of the *S. cerevisiae* TREX-2^ΔC^ complex

The TREX-2 complex obtained by Baculovirus expression was then used for structural analysis. Because of the size of the complex (195 kDa), a complementary approach was taken using electron microscopy (using both negative-staining and cryo-EM) together with crystallography and small-angle X-ray scattering.

**Fig. 2:**
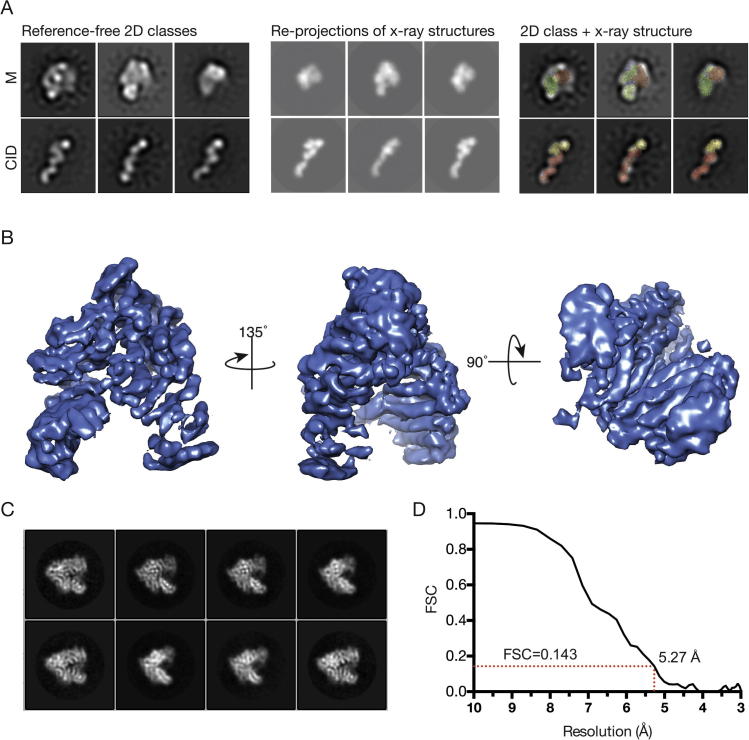
(A) Left panel: Three representative reference-free 2D class averages corresponding to the M- and CID-subcomplexes. Middle panel: Re-projections of the corresponding crystal structures in the approximate orientations as shown in the left panel. Right panel: superposition of the crystal structure over the reference-free 2D classes (Brown-Sac3, Green-Thp1, Blue-Sem1, Red-Sus1, Yellow-Cdc31). (B) Overview of the 5.3 Å cryo-EM reconstruction of the M-region of TREX-2. (C) Examples of eight class averages corresponding to the M-subcomplex from the cryo-EM data. (D) Gold-standard FSC curve for the 3D density map after RELION post-processing. The resolution is estimated to be 5.3 Å by the gold-standard refinement criterion, as indicated by the dotted red line.

Electron micrographs of the *S. cerevisiae* TREX-C^ΔC^ complex negatively stained with uranyl acetate showed two different motifs that were classified using reference-free 2D classification methods as implemented in Relion-1.4 (Fig. 2A). Class averages of one motif (Fig. 2A left panel, upper) had a V-shaped appearance and resembled the structure of the M-complex obtained by crystallography (Ellisdon et al., 2012). The second motif observed was elongated and class averages (Fig. 2A left panel, lower) showed that it contained three bulges and resembled the structure of the CID domain in which Cdc31 and two Sus1 chains wrap around a ∼120 Å Sac3 α-helix (Jani et al., 2009). Visual comparisons of the re-projections of the crystal structures (low-pass filtered to 20 Å to match the negative-stain data) agree well with the reference-free 2D classes (Fig. 2A middle panel). However, we were unable to detect a consistent spatial relationship between both motifs, consistent with the CID region not having a defined orientation relative to the M region, and so both motifs had to be processed independently.

**Fig. 3:**
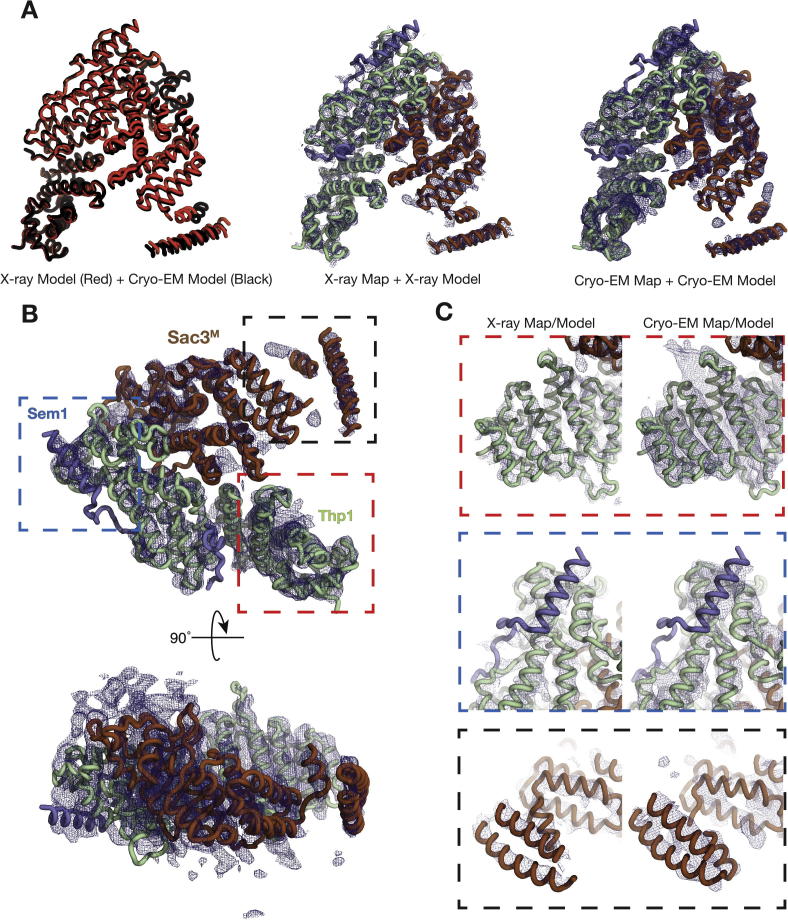
(A) The Cα-trace superposition of the model fitted based on the X-ray data (red) and the cryo-EM map (black) built based on the X-ray data (middle) and the cryo-EM map (right). Although there are minor perturbations in the map between Sac3 and Thp1, the overall structures are very similar and the “V”-shaped nature of the M-subcomplex is conserved. (B) Two views of the cryo-EM map fitted with the model built using the 3T5V crystal structure and poly-Ala helices for the additional density. (C) Comparisons of the two maps as boxed in panel B. It is clear that the region of Sac3 that was newly built (boxed in black) moves in the cryo-EM map relative to the X-ray map. Other helical features corresponding to Thp1 (boxed in red) and Sem1 (boxed in blue) agree well between the two maps.

Cryo-EM gave a substantial increase in resolution, but the signal/noise ratio of the images (as a consequence of the lower contrast and small mass of the object) frustrated identification of the complete CID complex and it was possible only to recognize the putative M-region (Fig. 2B). Analysis of 81,559 particles derived from 496 micrographs produced class averages (Fig. 2C) that enabled a 5.3 Å resolution reconstruction to be generated into which, despite problems arising from the objects exhibiting considerable preferred-orientation, it was possible to fit the crystal structure of the M-region complex using rigid body refinement (Table 1, Fig. 3 and Supplementary Fig. S4). The reconstruction showed clear electron density corresponding to the helices of the TPR-like repeats present in both Sac3 and Thp1 together with the winged-helix domain of each chain and also density corresponding to the part of Sem1 that also showed clear electron density by crystallography (Fig. 3B). In addition to giving an excellent fit to the crystal structure, the electron density from the cryo-EM reconstruction also showed three cylinders of density indicative of additional α-helices present in region of the Sac3 chain N-terminal to residue 253 at which the previous crystal structure terminated. There was however, no clear additional density that could be identified with the linkers between these helices or with the CID region of the complex, consistent with the CID region not having a defined orientation relative to the M-region as observed in the negatively-stained material. It is likely that within the cryo-EM micrographs recorded that the CID-subcomplex is also present in random orientations, however since the CID-subcomplex is effectively little more than a single α-helix with small proteins attached, the signal-to-noise is too poor for the particles to be detected with confidence (Supplementary Fig. S2).

Crystals with *P2_1_2_1_2_1_* symmetry with *a* = 78.2 Å, *b* = 84.1 Å, *c* = 165.5 Å that diffracted to 4.9 Å resolution using synchrotron radiation were obtained of the TREX-2 complex (Table 2). However, the volume of the asymmetric unit (1,087,701 Å^3^) would only have accommodated the entire complex if the solvent content was of the order of 12% and so it was likely that they instead contained a proteolytic fragment of the complex. Consistent with this hypothesis, limited proteolysis experiments showed that in the TREX-2 complex Sac3 was cleaved by both trypsin and chymotrypsin to yield a stable fragment at about ∼60 kDa (Supplementary Fig. S3). Phases were obtained using molecular replacement with the crystal structure of the M-subcomplex (PDB ID: 3T5V) and the electron density maps obtained after refinement and rebuilding were strikingly similar to those obtained by cryo-EM and showed the Sac3, Thp1 and Sem1 chains (Fig. 3C). The TPR-like repeat helices and the winged helix domains of both Sac3 and Thp1 were clear, although in places the density corresponding to some of the loops was less clear. Again there were three clear cylinders of additional density, consistent with α-helices, past residue 253 at which the original structural models (PDB accession codes 3T5V and 4TRQ) were truncated and was consistent with the Sac3 TPR-like repeat region extending further towards the N-terminus. Unfortunately it was not possible to identify unequivocally the residues linking these helices and so, because the density due to the side chains in these cylinders was not sufficiently clear to enable individual residues to be assigned unequivocally and so they were modeled as polyAla. No other difference density was observed that could correspond to the CID domain of the complex (containing Sac3 residues 727–805, together with two Sus1 chains and a Cdc31 chain), consistent with the crystals containing only the N-terminal half of Sac3 (up to about residue 555) together with Thp1 and Sem1. Although the material used in crystallization trials contained Sac3 residues 1–805, it was likely that, as is commonly observed, the CID-subcomplex and possibly also residues at the Sac3 N-terminus had been removed during the 2 weeks needed for the crystals to grow. Consistent with the possibility, micro-seeding attempts using these crystals in crystallization trials using freshly-prepared complex were unsuccessful.

In both negatively-stained and cryo-EM micrographs we observed additional density for the Sac3^M^ domain protruding from where the previous crystal structure started (residue 253). Overall the particles have a characteristic “V” shape and the additional Sac3 density was not in contact with Thp1. Since the additional density was consistent with TPR-like repeats, we have assigned this density to Sac3 N-terminal residues. Because these features were slightly blurred in the 2D-classes generated from the cryo-EM data (Fig. 2C), there might be some flexibility in this region of the protein, whereas in the crystals this region may be stabilized by crystal contacts. Unfortunately the 4.9 Å resolution electron density obtained from the current crystals was not sufficiently clear to enable unequivocal identification of the residues in the additional Sac3 cylinders seen both by cryo-EM and crystallography, but sequence analysis using the PSIPRED package (http://bioinf.cs.ucl.ac.uk/psipred – Buchan et al., 2013) indicated that residues 143–152, 154–177, 206–215, and 233–240 had a high potential for forming α-helices, whereas residues 1–142 showed little potential for forming secondary structure. We could not find additional density consistent with residues beyond 551 (connecting the Sac3 M-subcomplex and the Sac3 CID-subcomplex) and so it is likely that this linker is flexible, consistent with the random orientation observed for the CID region relative to the M-region in negatively stained electron micrographs.

### 3.3 Small-angle X-ray scattering

The electron microscopy and crystallography data was complemented by collecting small-angle X-ray (SAXS) data to assess the structure of the TREX-2 complex in solution (Table 3). We analyzed the full TREX-2 complex, the two subcomplexes (M- and CID-) and a construct of TREX-2 where the N-terminus of Sac3 had been deleted (TREX-2^140–805^) that was shown previously to display mRNA export and growth defects *in vivo* (Dimitrova et al., 2015). All constructs studied had calculated masses that agreed well with their respective theoretical masses, indicating that these complexes were primarily monomeric in solution. Although the CID-subcomplex (50.6 kDa) has only half the mass of the M-subcomplex (99.5 kDa), it displayed a similar radius of gyration, consistent with the CID-subcomplex having a rod-like shape compared to the M-subcomplex that is more globular. The TREX-2^140–805^ complex obtained by deleting Sac3 residues 1–140 exhibited a radius gyration of ∼7.6 nm which was greater than that exhibited by TREX-2^1–805^ (∼6.9 nm) consistent with deletion of Sac3 residues 1–140 generating a more extended conformation.

## 4 Discussion

Using complementary structural techniques we have investigated the structural properties of the *S. cerevisiae* TREX-2 complex using material expressed in Baculovirus to circumvent the difficulties experienced in obtaining large quantities of material from yeast cells. Although previous studies have suggested that Sac3 residues 220–250 are inherently disordered (Schneider et al., 2015), our results obtained by cryo-EM and crystallography point towards there being considerable secondary and tertiary structure in this region and indicate that there are three helices present in the Sac3 chain before residue 256. Thus, both our structures determined by X-ray crystallography and cryo-EM have demonstrated that Sac3^M^ continues to have TPR-like repeats before residue 252 and is likely to extend as far as residue ∼100. These extra TPR-like repeats do not make contact with Thp1 and from the slight blurring visible in the cryo-EM 2D classes might possibly have a degree of flexibility, which could potentially make them liable to proteolysis and so account for difficulties in observing electron density from this region in the previous work.

The Sac3^M^:Thp1:Sem1 region forms an interaction platform by which the TREX-2 complex interacts with both mRNAs and the mediator complex (Ellisdon et al., 2012, Schneider et al., 2015) and so the demonstration that the TPR-repeat region extends well below residue 255 indicates that the interaction interface between these components may be more extensive than previously thought. Although higher resolution structural data will be required to define this region precisely, it is interesting that deletion of Sac3 residues 1–140 generates both growth and mRNA export defects that are similar to those observed in a *SAC3* null strain (Dimitrova et al., 2015) indicating that this region is important for Sac3 function in the TREX-2 complex.

In contrast to previous work conducted with *C. thermophilum* TREX-2 complex (Dimitrova et al., 2015), our EM and crystallography data has suggested it is unlikely that the *S. cerevisiae* CID-subcomplex has a well-defined spatial orientation with respect to the M-subcomplex. However, it is still plausible that the Sac3^N^ region forms an interaction with the CID-subcomplex and therefore deletion of the first 140 residues of Sac3 introduces added degrees of freedom to the TREX-2 complex, thus increasing the radius of gyration that was observed in the SAXS measurements.

In summary, we describe here a method to recombinantly overexpress and purify the entire TREX-2 complex from *S. cerevisiae* using baculovirus infected sf9 cells. The availability larger quantities of the TREX-2 complex will facilitate experiments that have been previously difficult due to the lack of material. Complementary structural analysis of this material has demonstrated that in contrast to the previously characterized TREX-2 complex from *C. thermophilum* that the CID-subcomplex is likely not to have a defined spatial arrangement with respect to the M-subcomplex and that the TPR-like repeat region of the Sac3 chain extends further towards the N-terminus than had been previously thought.

## 5 Data deposition

The structure of the TREX-2 M-region determined by cryo-EM has been deposited in the Electron Microscopy Data Bank (accession No. EMD-3440) and PDB accession code 5G5P. Coordinates and diffraction data for the structures of the crystals of the TREX-2 complex M-region have been deposited in the Protein Data Bank with accession code 5L3T.

## Figures and Tables

**Table 1:** Cryo-EM Statistics.

Data collection	
Particles	81,559
Pixel size (Å)	1.43
Voltage (kV)	300
Defocus range (μm)	0.8–4.5
Electron dose (e-/Å^2^)	40

Model Statistics	
Molprobity score	1.32 (100th percentile)
All-atom clash score	5.79 (100th percentile)
Ramachandran Favoured/Outliers (%)	98.0/0

**Table 2:** Crystallography Statistics.

Synchrotron	DLS (Oxford, U.K.)
Beamline	I24
Collection Date	27/11/2015
Detector	Pilatus3 6M
Data reduction software (Version)	XDS
Data scaling software (Version)	AIMLESS (0.5.17)

Data collection statistics	
Wavelength (Å)	0.9686
Space group	*P*2_1_2_1_2_1_
Unit cell parameters: a, b, c (Å); α, β, γ (°)	78.2, 84.1, 165.5; 90.0, 90.0, 90.0
Resolution range (outer shell in brackets; Å)	47.08–4.90 (5.48–4.90)
Unique reflections	5268 (1437)
Total observations	24777 (6341)
<I/σ(I)>: all (outer shell)	4.0 (1.6)
R_p.i.m._: all (outer shell)	0.21 (0.64)
Completeness: all (outer shell) (%)	98.7 (97.5)
Multiplicity	4.7 (4.4)
Wilson B-factor	73.9

Refinement statistics	
Bond length deviation from ideal values (Å)	0.002
Bond angle deviation from ideal values (°)	0.49
Ramachandran favoured/outliers (%)	98.0/0.4
All-atom clashscore	4.9
Rwork/Rfree (%)	30.2 / 37.1

**Table 3:** SAXS Data.

	Calculated Mass (kDa)	Theoretical Mass (kDa)	Real Rg (nm)	Recip. Rg (nm)
Sac3^M^:Thp1:Sem1	100	99.5	3.43	3.43
Sac3^CID^:Cdc31:Sus1	48	50.6	3.28	3.28
TREX-2^1–805^	206	195	6.67	6.63
TREX-2^140–805^	190	180	7.66	7.61
