# Supplementary material for: The Sac3 TPR-like region in the Saccharomyces cerevisiae TREX-2 complex is more extensive but independent of the CID region
Source: J Struct Biol. 2016 Sep;195(3):316–24. doi: 10.1016/j.jsb.2016.07.007 (PMC4991852; doi:10.1016/j.jsb.2016.07.007)
Supplement: Supplementary data [file mmc1.pdf]

Supplementary Information for Expression, purification, and structural characterization of Recombinant TREX-2 complex from *Saccharomyces cerevisiae*

by Shintaro Aibara, Xiao-Chen Bai and Murray Stewart, MRC Laboratory of Molecular Biology, Francis Crick Avenue, Cambridge Biomedical Campus, Cambridge CB2 0QH

Supplementary Figures

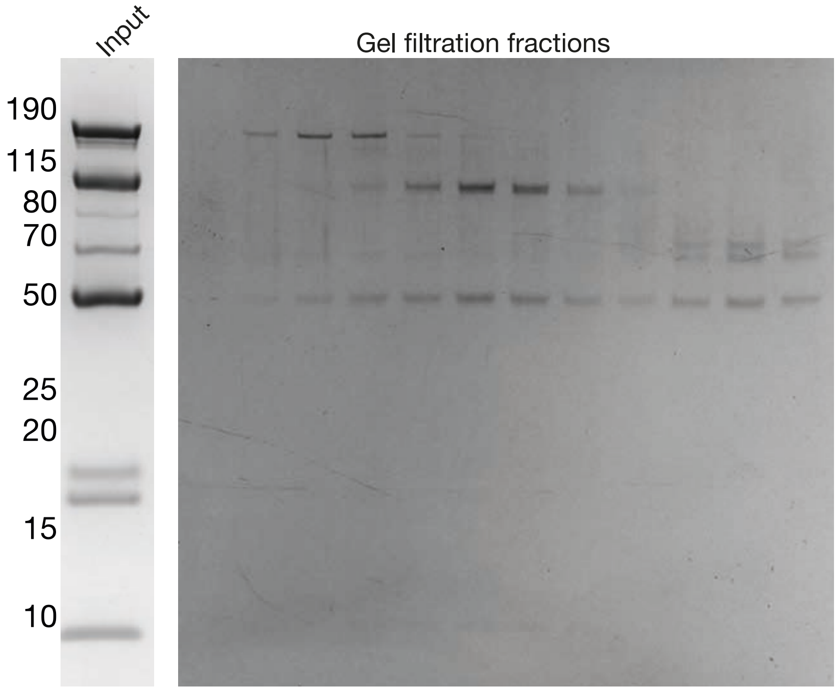

Supplementary Figure 1 Pooled and concentrated sample of full-length *sc*TREX-2 prior to loading onto gel filtration (left) and the eluted fractions (right). While some full-length Sac3 (~150 kDa) can be recovered from gel-filtration, it is clear that the protein suffers from proteolytic degradation.

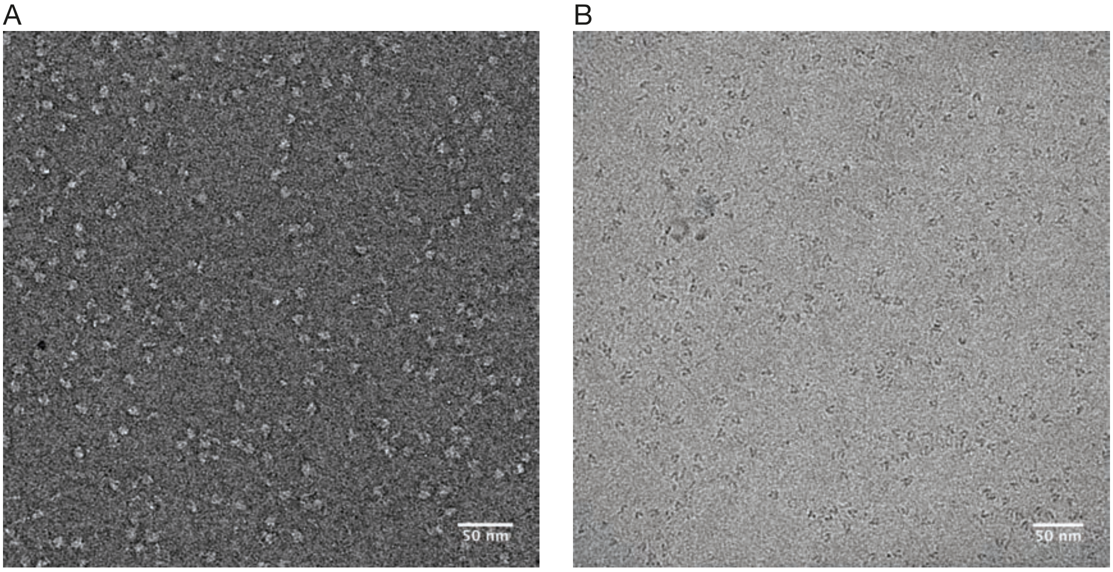

Supplementary Figure 2. Negatively stained (A) and cryo-EM (B) images of the *S. cerevisiae* TREX-2 complex expressed in Baculovirus.

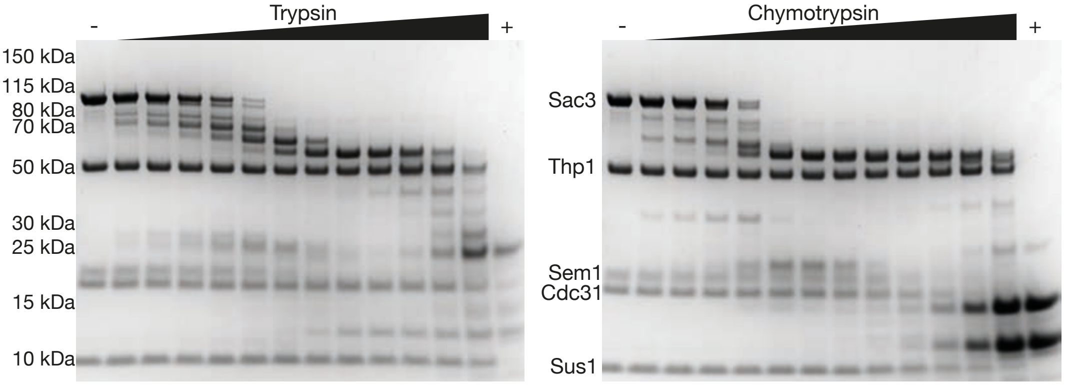

Supplementary Figure S3. Limited proteolysis of the S cerevisiae TREX-2 complex with either trypsin (A) or chymotrypsin (B) generates a ~60kD fragment of Sac3 that mass spectrometry confirmed contained peptides deriving from residues 1-555.

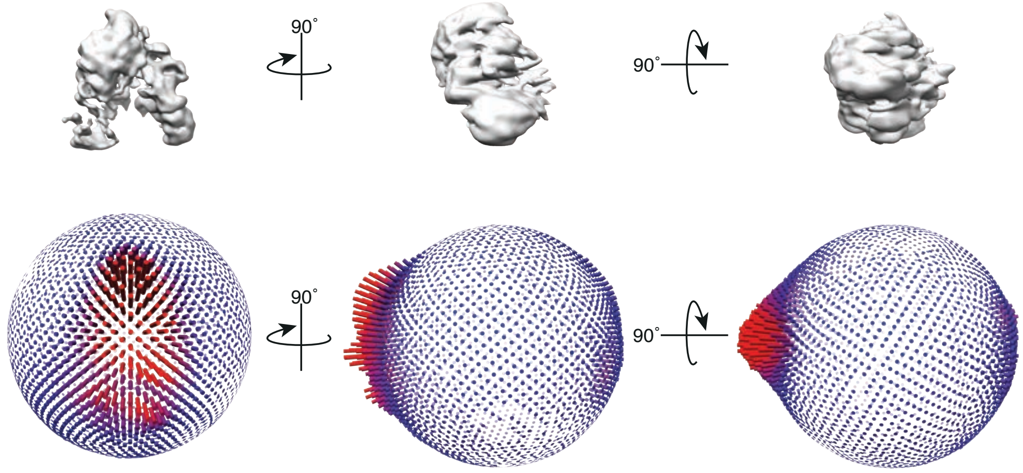

Supplementary Figure S4. Cryo-EM map of *sc*TREX-2 prior to post-processing (upper panel) and the corresponding visual representation of the angular distribution of particles as outputted by Relion (lower). Red rods represented frequent views whereas blue rods represent scarce views.
